# Spectral Engineering with Quantum Dot Films for Enhanced Crop Growth

**DOI:** 10.1021/acsaom.5c00338

**Published:** 2025-10-02

**Authors:** Kristine Q. Loh, Nathan J. Eylands, Vivian E. Ferry, Uwe R. Kortshagen

**Affiliations:** † Department of Chemical Engineering and Materials Science, 5635 University of Minnesota, Minneapolis, Minnesota 55455, United States; ‡ Department of Horticultural Science, 5635 University of Minnesota, St. Paul, Minnesota 55108, United States; § Department of Mechanical Engineering, 5635 University of Minnesota, Minneapolis, Minnesota 55455, United States

**Keywords:** Quantum Dots, Crop Growth, Spectral Shifting, Passive Spectral Manipulation, Sunlight Filter

## Abstract

Passive spectral manipulation strategies tune transmitted sunlight to more optimal wavelengths for plant growth. Quantum dots (QDs) embedded in polymer films are a promising material system for this application. Here, we simulate lettuce growth under nine different nontoxic QD films. QDs that strongly absorb blue/green light and downshift it to red/far-red wavelengths result in yield enhancements of up to 45%. We find that these QD films can be utilized broadly in greenhouses in the United States. Contrary to prevailing belief, increasing the intensity of down-shifted photoluminescence does not further increase yield, indicating that QD absorption is the most important factor.

## Graphical abstract

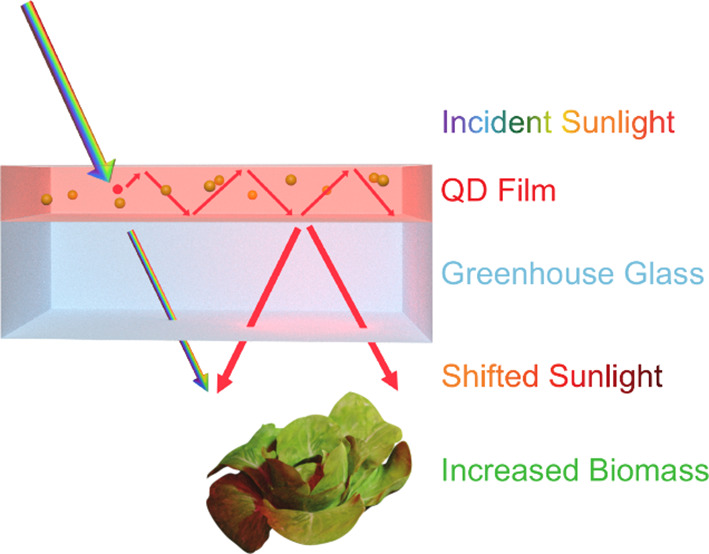

Light is one of the most important contributors to plant growth, as it is one of the primary drivers of photosynthesis.^1^ Horticultural lighting strategies have been investigated for a variety of crops because each cultivar requires different intensities and spectral distributions of light, opening opportunities for highly tailored, optimized agricultural practices.^2^ To enhance photosynthesis, spectral shifting or light conversion can be employed.^3,4^ This strategy maximizes photosynthetic efficiency by not only increasing the number of photons in the extended photosynthetically active radiation (ePAR; λ = 400 – 750 nm) range, but also by shifting sunlight into wavelength ranges more efficiently utilized by the plant. This extended range beyond 700 nm considers recent studies that indicate far-red light also contributes to crop growth.^5^

Greenhouses provide controlled environments for agricultural practices, but light management in these structures remains a challenge. The installation of spectral shifting films on greenhouses can take advantage of abundantly available sunlight to boost crop yields without the additional cost of supplementary lighting. Quantum dots (QDs) are semiconductor nanocrystals that have recently been investigated for their use in agricultural spectral shifting films^6^ because they absorb higher energy light and downshift it to lower energies through photoluminescence (PL).^7^ QDs are an attractive class of materials for the enhancement of biomass accumulation because their optical properties can be tailored by tuning their size and concentration. Their large and tunable Stokes shifts also allow for spectral separation of the PL from absorption and minimize reabsorption losses. Core/shell nanocrystals, for example, exhibit large effective Stokes shifts due to strength of absorption in the shell.^7^ Si QDs exhibit large Stokes Shifts due to their indirect bandgap.^8^ Furthermore, the myriad elemental compositions of QDs and their innumerable combinations have created a vast library of materials with absorption and PL spectra across and beyond the ePAR range.

Downshifting materials studied for plant growth include amorphous carbon dot films,^9^ glasses doped with perovskite nanocrystals,^10^ and films doped with fluorescent dyes.^11^ However, these materials are disadvantageous either due to a lack of tunable synthetic routes or a need for toxic elements, such as cadmium (Cd) and lead (Pb). To mitigate these concerns, copper indium sulfide core/zinc sulfide shell (CIS/ZnS) QD films have been developed. This elemental combination produces orange and red PL capable of increasing the yield of lettuce and sweet basil.^12^ This technology has also been commercialized for greenhouse films through the company UbiGro. Cd-based QD films have also been used to enhance the yield of cucumber, tomato, pumpkin, and pepper, but have yet to reach commercial scales due to concerns about the toxicity of Cd.^13^ Given the vast library of materials that could be used, general insight into the design of these passive, sunlight-shifting films is needed.

In this work, we deconvolute the complex design choices in QD films for agriculture, as QD selection, QD loading, and the extent of the PL outcoupling control the characteristics of irradiance that reaches the plant. We examine the QD film parameters that result in additional biomass accumulation for lettuce, a typical crop grown in controlled environment agriculture (CEA) whose response to spectral balance is still under investigation. In using lettuce as a model crop, we combined an optical model^14^ with a biomass accumulation model derived from a large set of experimental spectrum-dependent lettuce growth trials^15^ to isolate the influences of QD selection, loading, and PL outcoupling on their performance as agricultural sunlight filters. Light transmission through the QD films, assumed to be 150 μm thick throughout this study, primarily considered concentration-dependent absorption by the QDs as well as escaped PL from the bottom of the film. The lettuce model used in this work calculated the change in both structural and nonstructural dry weight over time considering light spectrum, light intensity, and plant temperature. QD films that strongly absorb blue and green light resulted in growth enhancements over 40% when compared to growth under the standard solar spectrum at equivalent intensities. Although QD films reduce transmitted light intensity, they could still be utilized across the continental United States; in spring and summer months, the films could reduce photoinhibition, or reduction in photosynthetic efficiency due to over lighting, and accelerate plant growth through spectral manipulation. Further yield improvements for the set of QDs studied in this work could not be achieved by increasing either the PL quantum yield or the film outcoupling efficiency, suggesting the QD absorption spectrum drives the influence of spectral shifting and therefore plant growth. These results support the use and future investigation of passive, spectral shifting QD films by providing insights into which QD film properties determine success in this application space.

Figure 1 depicts the absorbance and PL spectra, obtained from luminescent solar concentrator literature,^16–22^ of nine nontoxic QDs studied in this work. The QDs are organized into three categories: carbon dots, QDs with peak PL wavelengths in the visible range from 400 – 700 nm, and QDs with peak PL wavelengths in the near-infrared (NIR) range longer than 700 nm. The carbon dots were selected such that both the absorbance and PL spectra spanned the ePAR range. This allowed for blue-, green-, and red-emitting carbon dots which absorbed light in the ultraviolet (UV), blue, and green, respectively. The remaining QDs primarily absorb UV and blue light, with variances in the peak PL wavelength that depended on the QD elemental composition. The probability of a photon being emitted after absorption, or the PL quantum yields (PLQYs), for all QDs studied varied broadly from 30% to 92%, as listed in Table S1. Our selection not only avoids the common use of Cd and Pb in QDs, mitigating toxicity concerns, but also spans the wavelength range of interest for plant photochemistry.

**Figure 1:**
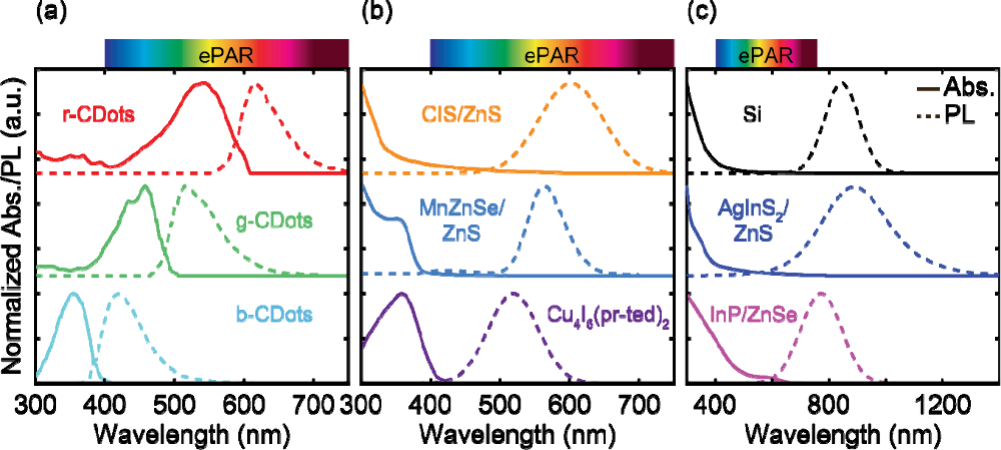
Absorption (solid lines) and photoluminescence (PL) spectra (dashed lines) for all quantum dots (QDs) studied, organized into (a) carbon dots, (b) QDs with PL in the visible range, and (c) QDs with PL in the near-infrared range (λ > 700 nm).^16–22^ Extended photosynthetically active radiation (ePAR; λ = 400 – 750 nm) ranges are highlighted in rainbow.

Figure 2 demonstrates the impact of QD film concentration on simulated lettuce yield. Increasing the concentration of the QD film increases absorption by the QDs, thus decreasing the intensity of transmitted light in the spectral range of absorption. However, some of the down-shifted PL by the QDs escapes out of the film’s bottom surface, increasing the amount of longer-wavelength light that the plants receive. To control for light intensity, we set the number of photosynthetic photons available to the plants over the course of a day, or the extended daily light integral (eDLI), at a constant 19.2 mol m^−2^ d^−1^, in alignment with optimal light requirements for growing lettuce.^1^ Considering both transmitted light through the film and down-shifted PL, we controlled the eDLI such that the integrated spectral intensity was the same across all QD concentrations. More details regarding these calculations are provided in the Supporting Information. With a constant eDLI, the influence of the different color spectra on crop yield can be directly studied.

**Figure 2:**
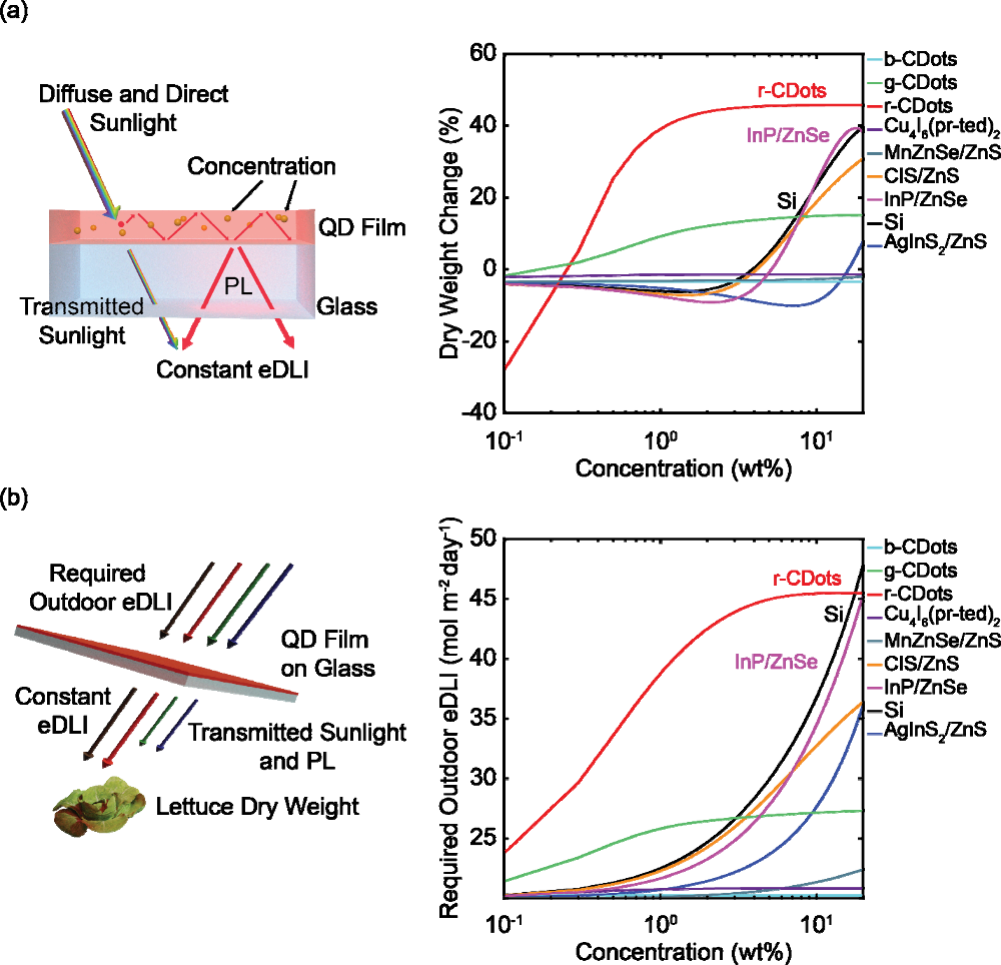
(a) Schematic of control over the extended daily light integral (eDLI) through a spectral shifting QD film with 150 μm thickness to demonstrate the dependent and independent variables. The change in dry weight of lettuce grown under QD films compared to that grown under the solar spectrum (12.5 g) is a function of quantum dot (QD) selection and concentration. (b) Schematic of the required outdoor eDLI to meet a constant eDLI of 19.2 mol m^−2^ d^−1^ for plant growth. With increasing concentration, the required outdoor eDLI increases up to 45 mol m^−2^ d^−1^, depending on QD selection. QD films that resulted in the greatest yield enhancements (r-CDots, Si, and InP/ZnSe) have additional labels.

Figure 2(a) illustrates how varying the concentration in all nine QD films from 0.1 to above 10 wt %, resulted in changes to the transmitted sunlight, thus resulting in changes to lettuce yield. Compared to lettuce grown under the solar spectrum at the same integrated light intensity, lettuce grown under r-CDots, InP/ZnSe, and Si films at concentrations above 10 wt % were predicted to have the greatest yield enhancement, from 20%–45%. These enhancements corresponded to changes in the dry weight from 12.5 g up to 18.2 g per plant, as shown in Figure S4. This increase was due to the relative reduction of blue and green light fractions, with the accompanying increase in red and far-red light fractions. Blue- and green-emitting QDs, such as b-CDots and Cu_4_I_6_(pr-ted)_2_, did not significantly change lettuce yield. Similarly, QDs with PL outside of the ePAR range, such as AgInS_2_/ZnS, did not significantly impact crop yield. These results demonstrate that, when prioritizing crop growth, enhancing red and far-red light while reducing blue and green light transmission will increase yield compared to growth under the unaltered solar spectrum.

Because high-concentration QD films may significantly reduce transmission, we then determined the required intensity of light incident on the film to reach the target eDLI at the plant canopy. Figure 2(b) demonstrates the significant reduction in transmission at high QD concentrations for some QDs. For example, for r-CDot films at concentrations above 1 wt %, less than 50% of sunlight is transmitted, requiring incident eDLIs over 40 mol m^−2^ d^−1^ to meet the optimal light requirement. Furthermore, UV radiation comprises 8% of the AM1.5G solar spectrum (W m^–2^ nm^–1^) in the range of 300–750 nm. QDs that only absorb sunlight in the UV range, such as MnZnSe/ZnS, b-CDots, or Cu_4_I_6_(pr-ted)_2_, transmit more radiation, but also result in limited yield improvements. Overall, these results indicate that the best-performing QD films that more strongly absorb blue and green light must be used in climates with at least 30 mol m^−2^ d^−1^ of incident sunlight to achieve sufficient yield enhancements.

To confirm whether Cd- and Pb-containing QDs could further increase lettuce yields, we conducted similar calculations for eight QDs with these elements. Figure S5 depicts their absorption and PL spectra, also obtained from luminescent solar concentrator literature.^23–28^ Figure S6 illustrates the resulting changes in dry weight as well as the required outdoor eDLIs. Because only one QD option (6 wt % CdSe/CdS) warranted the consideration of Cd- and Pb-containing films, crops would not further benefit from the implementation of these more commonly studied toxic QDs.

To determine where high-concentration QD films could be utilized without reducing light transmission below 19.2 mol m^−2^ d^−1^, we retrieved DLI values across the continental United States^29^ then recalculated them as eDLIs to incorporate far-red light. If the QD films were implemented in CEA structures, such as a greenhouse, the framing structures must be considered as obstacles to light transmission as well. We assumed greenhouse framing blocked 20% of incident sunlight.^30^ More details regarding this conversion are provided in the Supporting Information. To consider the stability of nontoxic QD films over the time scale of a harvest cycle, we refer to work by Terricabres-Polo et al., who demonstrated the photostability of InP/ZnSe/ZnS and CuInS_2_/ZnS films upon incorporation into a photoactive block polymer.^31^ These nontoxic QD films were stable over two years in outdoor conditions, encompassing multiple potential growth cycles for lettuce. Figure 3 illustrates the eDLI for plants in greenhouses across the continental United States throughout the year. In alignment with the sunlight requirements shown in Figure 2(b), greenhouses located below roughly 40 °N from March through September experience at least 30 mol m^−2^ d^−1^ of sunlight. Therefore, these passive QD films could be utilized to support plant growth without the additional cost of supplementary lighting in these months. In the winter when the outdoor eDLI is below 30 mol m^−2^ d^−1^, supplementary lighting would be needed to meet minimum eDLI requirements for growing marketable yields.

**Figure 3:**
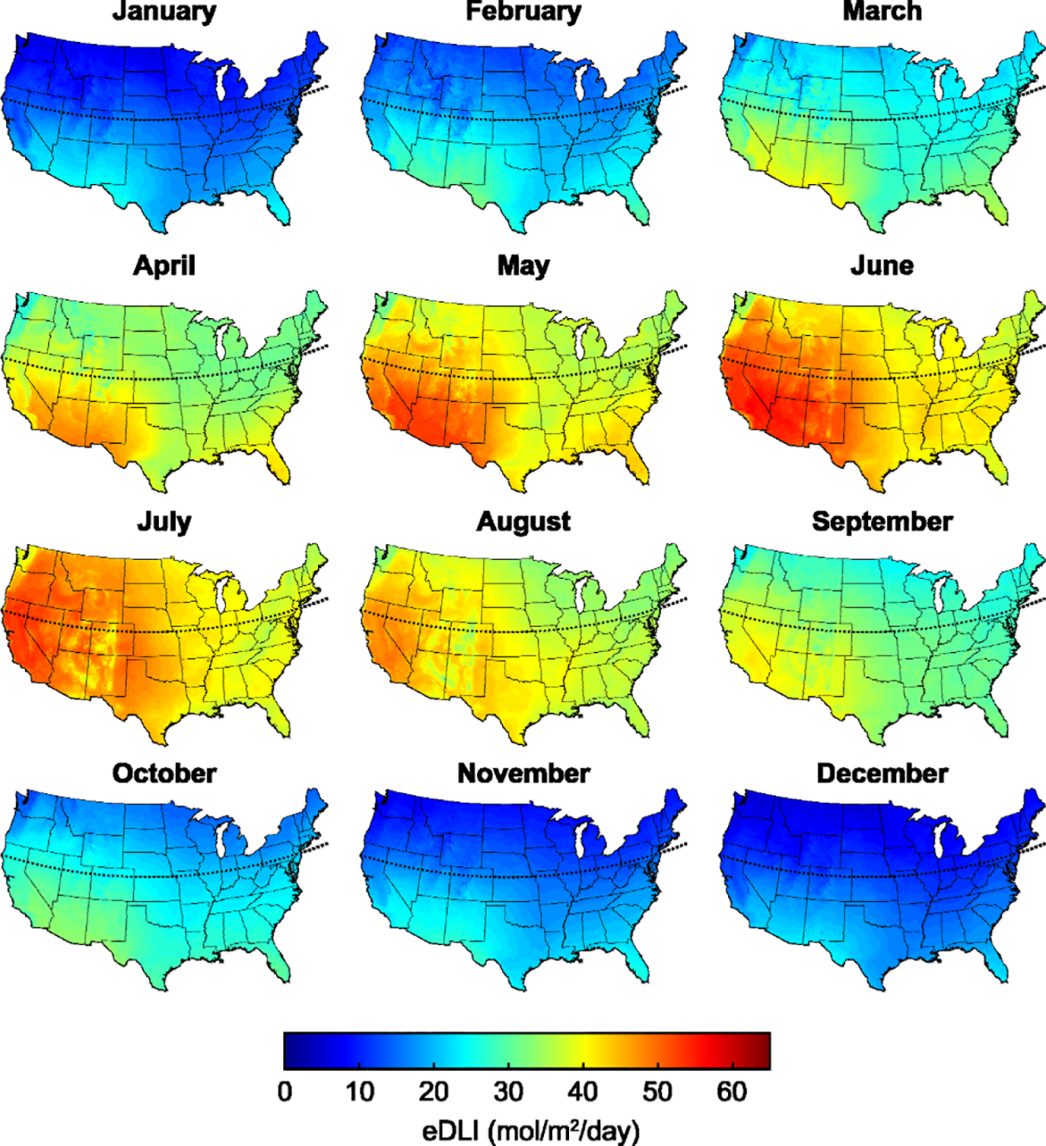
Extended daily light integral across the United States throughout the year, assuming the greenhouse framing blocks 20% of incident light. Dotted lines indicate 40 °N latitude lines for reference.

However, Figure S7 demonstrates that even at low constant eDLI values of 5 and 10 mol m^−2^ d^−1^, using QD films can further increase crop yield compared to growth under the unaltered solar spectrum. At a constant eDLI of 5 mol m^−2^ d^−1^, lettuce yields increased by over 650% from a dry weight of 0.63 g, and at a constant eDLI of 10 mol m^−2^ d^−1^, yields increased by over 125% from 5.2 g for crops grown under r-CDot films. This suggests that even in the winter at low outdoor eDLI, some QD films may be able to reduce the need for supplementary lighting. Although the changes in dry weight were significantly higher than those shown in Figure 2(a), it is important to note that lettuce yields under the unaltered solar spectrum were very low due to low eDLI values.

To explore whether the impact of spectral shifting was absorption-driven or PL-driven, the latter being the prevailing narrative, we determined the influence of QD PL intensity, which depends both on PLQY and film outcoupling efficiency, on the change in dry weight. Multiple strategies to reduce nonradiative losses in QDs and thus increase the PLQY have been investigated recently, including surface passivation, optimized processing conditions, and careful selection of the precursor materials.^7^ Most common polymers have a refractive index of 1.5; when placed in air, the refractive index mismatch causes around 75% of re-emitted PL from the QD to remain trapped in the waveguide. As a result, only 12.5% of emitted PL escapes from the bottom of the film. Strategies to increase the film outcoupling efficiency to disrupt the film’s total internal reflection modes include microcone or microdome arrays, refractive index engineering using photonic crystals, and nanoarrays of plasmonic materials.^32^ Given these trends, we sought to understand whether these improvements to light management could subsequently enhance crop yield.

Figure 4(a)–(c) demonstrates the limited impact of increasing the PLQY on improving crop yield when controlling for eDLI. The change in lettuce dry weight varied by around 5% when varying the PLQY from 0% to 100% while the PL outcoupling efficiency was kept at 12.5%. These results indicate that even if the QDs had no nonradiative losses and were perfectly efficient in downshifting light, the plants underneath the QD film did not significantly benefit from the increased fraction of red/far-red photons.

**Figure 4:**
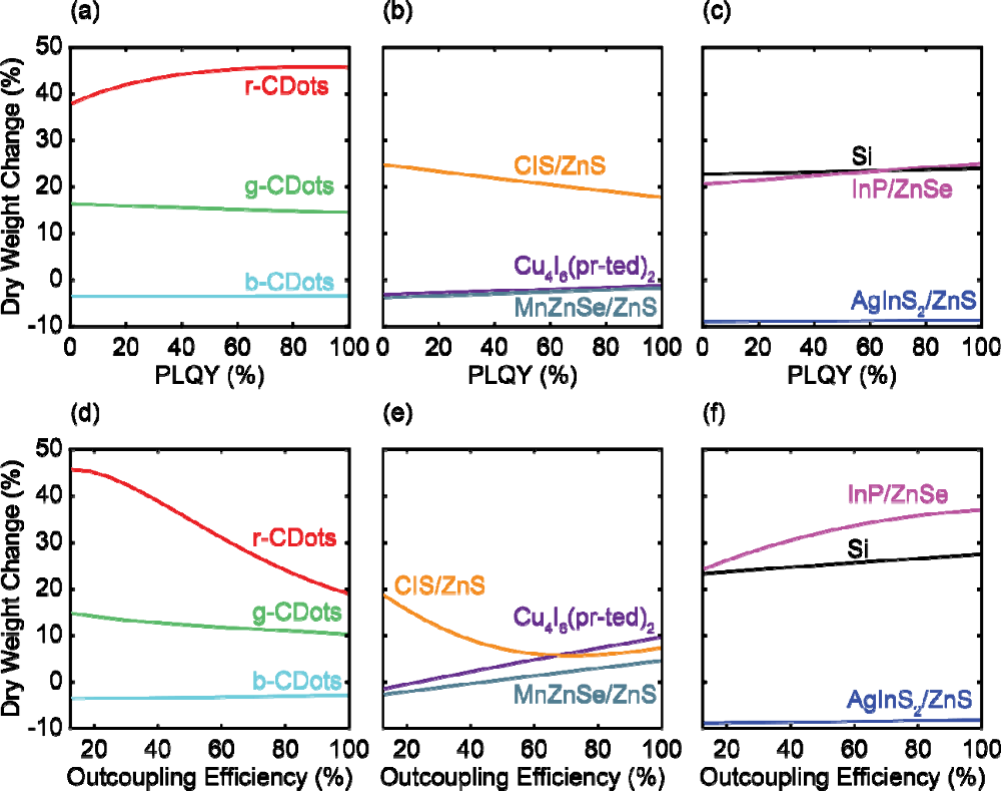
Change in dry weight in a 10 wt % film as a function of the photoluminescent quantum yield (a)–(c) and as a function of the film outcoupling efficiency (d)–(f). (a) and (c) show the changes in dry weight for carbon dot films; (b) and (e) show the changes in dry weight for quantum dot films with visible photoluminescence, and (c) and (f) show the changes in dry weight for quantum dot films with near-infrared photoluminescence.

We then changed the outcoupling efficiency while keeping the PLQY for each QD as the literature-based value listed in Table S1. However, Figure 4(d)–(f) indicates that further yield enhancements were not achieved. Increasing the film outcoupling efficiency from the bottom of the film from 12.5% to 100% for all QDs did not result in improved crop yield. QDs with PL spectra that overlapped multiple color ranges showed differing trends when varying outcoupling efficiency. For CIS/ZnS films, increasing the outcoupling efficiency increased the green light fraction, resulting in reduced biomass. InP/ZnSe films produced more biomass with increasing outcoupling efficiency due to increased red and far-red light fractions. Interestingly, increasing the outcoupling efficiency for r-CDot films decreased the calculated biomass, and for Cu_4_I_6_(pr-ted)_2_ films, the biomass increased.

Figure S8 illustrates the change in quantum use efficiency as a function of color fraction for a better understanding of these seemingly counterintuitive results. In the case of r-CDots films that strongly absorb green light, red fractions beyond 0.7 resulted in a decreased quantum use efficiency. For Cu_4_I_6_(pr-ted)_2_ films that do not strongly absorb sunlight, increasing the blue light fraction when all color fractions are roughly even, slightly increases the quantum use efficiency. Using the crop growth model created by Abedi et al., lettuce yield is optimized when the transmitted red light fraction is at most 0.7.^15^ One experimental investigation of lettuce grown under artificial lights in plant factories suggested the optimal blue:green:red distribution of 0.18:0.10:0.72, further expressing that growing lettuce under more red light than the proposed optimum does not increase yield.^33^ Figure 4 cumulatively demonstrates that the QD absorption spectrum was the primary driver for increasing crop yield. The design of QD films should thus prioritize the strong absorption of primarily blue and green light for higher crop yields.

Spectral effects on plant biomass gain posit two explanations: downshifting light created an altered spectrum available to the crop canopy that 1) enhanced photosynthetic efficiency or 2) increased radiation capture efficiency. The first postulate, photosynthetic efficiency, is a ratio that describes a plant’s ability to fix atmospheric CO_2_ on a per quantum basis.^34^ Blue and red photons display high absorptance in photosynthetic pigments and are predominantly absorbed by the top few layers of cells in leaves.^35^ On their own, these photons result in high quantum yield of CO_2_ assimilation in plants,^36^ but in broad spectrum studies under high photon flux, blue photons exhibit lower photosynthetic efficiency.^37^ The latter, and most likely postulate, is based on photomorphogenesis: plant development guided by light quality. Generally, plants grown under more blue light are shorter and have thicker leaves. Increasing blue light fraction inhibits cell division and expansion and therefore reduces leaf area.^38^ Reduced leaf area reduces a plant’s ability to capture radiation thereby reducing crop biomass gain.

The results of this work demonstrate the ability of nontoxic QD films to promote lettuce growth. QDs that absorb blue/green light while downshifting it to red and far-red radiation resulted in the greatest yield improvements. These films can be deployed across the continental United States throughout the year, resulting in additional biomass accumulation even if they cannot transmit sufficient light to meet optimal eDLI requirements in the winter. Further optimization of PLQY and outcoupling efficiency did not improve biomass accumulation beyond 45% at a constant eDLI of 19.2 mol m^−2^ d^−1^, suggesting that future research efforts should instead prioritize the engineering of QD absorption instead of PL. In addition to shaping transmitted light for optimized crop growth, QD films have another potential benefit of solar energy conversion for renewable electricity generation in the form of photovoltaic luminescent solar concentrators.^39^ PL that does not escape the film could be waveguided to attached solar cells, allowing for simultaneous benefits of both energy generation and crop production. To broaden the community’s understanding of how QDs can meet plant-specific light requirements, future studies on other crop types that may benefit from down-shifted PL as well as the influence of down-shifted PL on crop nutritional content and morphology should be conducted.

## Supplementary Material



## Data Availability

The data that support the findings of this study are openly available in DRUM at https://doi.org/10.13020/kafz-yc82.

## Notes

Simulation methods, dry weight of lettuce grown under the standard solar spectrum and transmitted spectra under QD films, absorption and PL spectra of Cd- and Pb-containing QDs, change in dry weight under Cd- and Pb-containing QD films with corresponding required outdoor eDLI, change in dry weight at low eDLI, and quantum use efficiency as a function of color fraction (PDF)

## Abbreviations

ePAR: extended photosynthetically active radiation.
eDLI: extended daily light integral.
NIR: near-infrared.
PLQY: photoluminescence quantum yield.
QD: quantum dot.
UV: ultraviolet.

## References

[1] Kelly N., Choe D., Meng Q., Runkle E. S. (2020). Promotion of Lettuce
Growth under an Increasing Daily Light Integral Depends on the Combination
of the Photosynthetic Photon Flux Density and Photoperiod. Sci. Hortic..

[2] Mishra K., Stanghellini C., Hemming S. (2023). Technology and Materials
for Passive
Manipulation of the Solar Spectrum in Greenhouses. Adv. Sustain. Syst..

[3] Ma
Lu S., Amaducci S., Gorjian S., Haworth M., Hägglund C., Ma T., Zainali S., Campana P. E. (2024). Wavelength-Selective Solar Photovoltaic
Systems to Enhance Spectral Sharing of Sunlight in Agrivoltaics. Joule.

[4] Loh, K. Q.-T. Nontoxic Nanomaterials for Luminescent Solar Concentrators in Agrivoltaic Systems. Ph.D. dissertation, University of Minnesota, 2025 https://www.proquest.com/docview/3234927280/abstract/733B89D0B66B4E32PQ/1 (accessed 2025–09–17).

[5] Pazuki A., Aflaki F., Pessarakli M., Gurel E., Gurel S. (2017). Plant Responses
to Extended Photosynthetically Active Radiation (EPAR). Adv. Plants Agric. Res..

[6] Khan Q., Wang A., Li P., Hu J. (2025). Quantum Dots Illuminating
the Future of Greenhouse Agriculture. Adv. Sustain.
Syst..

[7] Pietryga J. M., Park Y.-S., Lim J., Fidler A. F., Bae W. K., Brovelli S., Klimov V. I. (2016). Spectroscopic and Device Aspects
of Nanocrystal Quantum Dots. Chem. Rev..

[8] Meier C., Gondorf A., Lüttjohann S., Lorke A., Wiggers H. (2007). Silicon Nanoparticles:
Absorption, Emission, and the Nature of the Electronic Bandgap. J. Appl. Phys..

[9] Ge M., Yuan Y., Liu S., Li J., Yang C., Du B., Pang Q., Li S., Chen Z. (2024). Enhancing Plant Photosynthesis
with Dual Light Conversion Films Incorporating Biomass-Derived Carbon
Dots. Carbon Capture Sci. Technol..

[10] Chen Y., Shen L., Liu J., Liang X., Xiang W. (2021). Eco-Friendly
Mn-Doped CsPbCl3 Perovskite Nanocrystal Glass with Blue-Red Emission
for Indoor Plant Lighting. J. Am. Ceram. Soc..

[11] Stallknecht E. J., Runkle E. S. (2025). An Experimental
Red Fluorescent Film Has Cultivar-Specific
Effects on Lettuce Yield and Morphology. HortScience.

[12] Kang S., Parrish C. H., Hebert D., Zhen S. (2024). Luminescent Quantum
Dot Films Increase the Radiation Capture and Yield of Lettuce and
Sweet Basil Compared to a Traditional/Neutral-Density Greenhouse Glazing. HortScience.

[13] Ivanyuk V. V., Shkirin A. V., Belosludtsev K. N., Dubinin M. V., Kozlov V. A., Bunkin N. F., Dorokhov A. S., Gudkov S. V. (2020). Influence of Fluoropolymer
Film Modified With Nanoscale Photoluminophor on Growth and Development
of Plants. Front. Phys..

[14] Sychugov I. (2019). Analytical
Description of a Luminescent Solar Concentrator. Optica.

[15] Abedi M., Tan X., Stallknecht E. J., Runkle E. S., Klausner J. F., Murillo M. S., Bénard A. (2023). Incorporating the Effect of the Photon
Spectrum on Biomass Accumulation of Lettuce Using a Dynamic Growth
Model. Front. Plant Sci..

[16] Hill S. K. E., Connell R., Held J., Peterson C., Francis L., Hillmyer M. A., Ferry V. E., Kortshagen U. (2020). Poly­(Methyl
Methacrylate) Films with High Concentrations of Silicon Quantum Dots
for Visibly Transparent Luminescent Solar Concentrators. ACS Appl. Mater. Interfaces.

[17] Parrish C. H., Hebert D., Jackson A., Ramasamy K., McDaniel H., Giacomelli G. A., Bergren M. R. (2021). Optimizing Spectral Quality with
Quantum Dots to Enhance Crop Yield in Controlled Environments. Commun. Biol..

[18] Zdražil L., Kalytchuk S., Holá K., Petr M., Zmeškal O., Kment Š., Rogach A. L., Zbořil R. (2020). A Carbon Dot-Based
Tandem Luminescent Solar Concentrator. Nanoscale.

[19] Chen J., Zhao H., Li Z., Zhao X., Gong X. (2022). Highly Efficient
Tandem Luminescent Solar Concentrators Based on Eco-Friendly Copper
Iodide Based Hybrid Nanoparticles and Carbon Dots. Energy Environ. Sci..

[20] Sadeghi S., Bahmani Jalali H., Srivastava S. B., Melikov R., Baylam I., Sennaroglu A., Nizamoglu S. (2020). High-Performance, Large-Area, and
Ecofriendly Luminescent Solar Concentrators Using Copper-Doped InP
Quantum Dots. iScience.

[21] Chen W., Li J., Liu P., Liu H., Xia J., Li S., Wang D., Wu D., Lu W., Sun X. W., Wang K. (2017). Heavy Metal Free Nanocrystals with
Near Infrared Emission Applying
in Luminescent Solar Concentrator. Sol. RRL.

[22] Erickson C. S., Bradshaw L. R., McDowall S., Gilbertson J. D., Gamelin D. R., Patrick D. L. (2014). Zero-Reabsorption
Doped-Nanocrystal
Luminescent Solar Concentrators. ACS Nano.

[23] Zhou Y., Benetti D., Fan Z., Zhao H., Ma D., Govorov A. O., Vomiero A., Rosei F. (2016). Near Infrared, Highly
Efficient Luminescent Solar Concentrators. Adv.
Energy Mater..

[24] Connell R., Keil J., Peterson C., Hillmyer M. A., Ferry V. E. (2019). CdSe/CdS–Poly­(Cyclohexylethylene)
Thin Film Luminescent Solar Concentrators. APL
Mater..

[25] Li H., Wu K., Lim J., Song H.-J., Klimov V. I. (2016). Doctor-Blade Deposition
of Quantum Dots onto Standard Window Glass for Low-Loss Large-Area
Luminescent Solar Concentrators. Nat. Energy.

[26] Zhao H., Zhou Y., Benetti D., Ma D., Rosei F. (2017). Perovskite
Quantum Dots Integrated in Large-Area Luminescent Solar Concentrators. Nano Energy.

[27] Brennan L. J., Purcell-Milton F., McKenna B., Watson T. M., Gun’ko Y. K., Evans R. C. (2018). Large Area Quantum Dot Luminescent
Solar Concentrators
for Use with Dye-Sensitised Solar Cells. J.
Mater. Chem. A.

[28] Sumner R., Eiselt S., Kilburn T. B., Erickson C., Carlson B., Gamelin D. R., McDowall S., Patrick D. L. (2017). Analysis of Optical
Losses in High-Efficiency CuIn_2_-Based Nanocrystal Luminescent
Solar Concentrators: Balancing Absorption versus Scattering. J. Phys. Chem. C.

[29] Faust J. E., Logan J. (2018). Daily Light Integral:
A Research Review and High-Resolution Maps
of the United States. HortScience.

[30] Albright L. D., Both A.-J., Chiu A. J. (2000). Controlling Greenhouse Light to a
Consistent Daily Integral. Trans. ASAE.

[31] Terricabres-Polo R., de Bruin T. A., Kaul A., van Sark W. G. J. H. M., Donega C. de M. (2024). Durable Quantum Dot-Based Luminescent Solar Concentrators
Enabled by a Photoactive Block Copolymer. Adv.
Energy Mater..

[32] Timmermans G. H., Hemming S., Baeza E., van Thoor E. A. J., Schenning A. P. H. J., Debije M. G. (2020). Advanced Optical
Materials for Sunlight Control in Greenhouses. Adv. Opt. Mater..

[33] Razzak Md. A., Asaduzzaman Md., Tanaka H., Asao T. (2022). Effects of Supplementing
Green Light to Red and Blue Light on the Growth and Yield of Lettuce
in Plant Factories. Sci. Hortic..

[34] Bugbee B. (2016). Toward an
Optimal Spectral Quality for Plant Growth and Development: The Importance
of Radiation Capture. Acta Hortic..

[35] Brodersen C. R., Vogelmann T. C. (2010). Do Changes
in Light Direction Affect Absorption Profiles
in Leaves?. Funct. Plant Biol..

[36] McCree K. J. (1971). The Action
Spectrum, Absorptance and Quantum Yield of Photosynthesis in Crop
Plants. Agric. Meteorol..

[37] Liu J., van Iersel M. W. (2021). Photosynthetic
Physiology of Blue, Green, and Red Light:
Light Intensity Effects and Underlying Mechanisms. Front. Plant Sci..

[38] Dougher T. A. O., Bugbee B. (2004). Long-Term Blue Light Effects on the
Histology of Lettuce
and Soybean Leaves and Stems. J. Am. Soc. Hortic.
Sci..

[39] Purcell-Milton F., Gun’ko Y. K. (2012). Quantum Dots for Luminescent Solar
Concentrators. J. Mater. Chem..

