# Supplementary material for: Spectral Engineering with Quantum Dot Films for Enhanced Crop Growth
Source: ACS Appl Opt Mater. 2025 Oct 2;3(10):2251–6. doi: 10.1021/acsaom.5c00338 (PMC12560175; doi:10.1021/acsaom.5c00338)
Supplement: Supplementary file 1 [file ot5c00338_si_001.pdf]

Spectral Engineering with Quantum Dot Films for Enhanced Crop Growth

Supporting Information

Kristine Q. Loh,[†] Nathan J. Eylands,[‡] Vivian E. Ferry,[*,†] and Uwe R. Kortshagen[*,¶]

†*Department of Chemical Engineering and Materials Science, University of Minnesota, Minneapolis, Minnesota, 55455, United States*

‡*Department of Horticultural Science, University of Minnesota, St. Paul, Minnesota, 55108, United States*

¶*Department of Mechanical Engineering, University of Minnesota, Minneapolis, Minnesota, 55455, United States*

* E-mail: veferry@umn.edu

* Email korts001@umn.edu

Light Transmission Model

Absorbance and photoluminescence (PL) spectra were first retrieved from the literature to then calculate the light transmitted through a QD film.

Data Extraction

To obtain absorption coefficients for each of the nine nontoxic QDs in this work, the absorbance spectra were first retrieved using a web-based data extraction tool.[1] If axis limits

were not provided in the figure, it was assumed that the absorbance at the peak absorbance wavelength was equal to 1. The absorption coefficient in units of $\frac{1}{m\,wt\%}$ was then calculated as:

$$\alpha(\lambda) = \frac{\ln(10)A(\lambda)}{\tau_0 c_0} \tag{1}$$

where $A(\lambda)$ is the wavelength-dependent absorbance extracted from the literature, τ_0 is the thickness of the film in units of m, and c_0 is the concentration of the film in units of wt%.

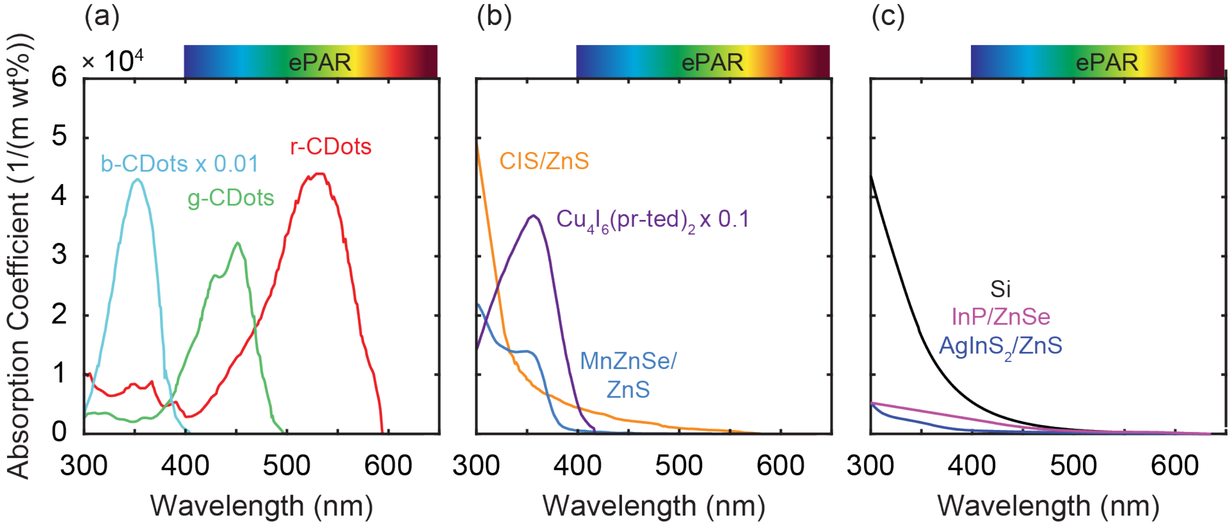

Figure S1: Absorption coefficients for the nine nontoxic QDs studied in this work in units of 1/(m wt%), grouped into (a) carbon dots, (b) QDs with visible PL, and (c) QDs with PL outside of the visible range. Absorption coefficients for b-CDots and Cu_4I_6(pr-ted)$_2$ were scaled.

Both the absorption coefficients and the PL spectra were interpolated to encompass the wavelength range of 300 to 2000 nm. The absorption coefficients assumed for each of the nine QDs used in this work are shown in Figure S1. A summary of the data extracted from the literature for this work, including the PL quantum yield (PLQY), original film thickness, original film concentration, and QD size are presented in Tables S1 and S2.

Table S1: Parameters used to determine absorption coefficients of the nine nontoxic QDs studied in this work, as well as their PL and size data.

QD	PLQY (%)	Thickness (μm)	Concentration (wt%)	Size (nm)
b-CDots[2]	63	50	0.0101	6.3
g-CDots[2]	78	100	0.294	2.9
r-CDots[2]	77	80	0.620	2.9
CIS/ZnS[3]	85	350	0.400	4.8
MnZnSe/ZnS[4]	78	50	0.195	~6
$Cu_4I_6(pr\text{-}ted)_2$[5]	92	10	0.600	3.18
Si[6]	50	83	0.813	3.1
$AgInS_2/ZnS$[7]	30	30,000	0.0137	~6
InP/ZnSe[8]	81	100	2.62	8.5

Table S2: Parameters used to determine absorption coefficients of the eight Cd- and Pb-containing QDs studied in this work, as well as their PL and size data.

QD	PLQY (%)	Thickness (μm)	Concentration (wt%)	Size (nm)
$CsPb(Br_2I_8)_{0.3}$[9]	63	2000	1.6	12.9
$CsPbBr_3$[9]	90	2000	1.6	8.5
PbS/CdS[10]	50	2000	0.00014	1.5
CdSe/CdS[11]	75	0.35	0.18	8.4
$CdSe/Cd_{0.6}Zn_{0.4}S$[12]	70	50	5.1	4
$Mn^{2+}Cd_{0.5}Zn_{0.5}S/ZnS$[13]	78	50	0.20	3
CIS/CdS[14]	45	1000	3.0	3-4
CdSe/ZnS[15]	65	2500	0.15	12.9

Transmission Calculation

First, the wavelength-dependent absorption probability of the QDs was calculated using Beer's Law:

$$P_{abs} = 1 - \exp(-\alpha(\lambda)c\tau) \tag{2}$$

where α is the the absorption coefficient, c is the concentration of QDs in the film, and τ is the thickness of the film (150 μm). The absorption probability was then weighted by the

solar spectrum:

$$A(\lambda) = P_{abs}F(\lambda) \tag{3}$$

where $F(\lambda)$ is the solar spectrum in units of $\frac{photons}{m^2 \cdot s}$ and $A(\lambda)$ is the wavelength-dependent absorbance of the QDs. Normal incidence was assumed for this work. The emitted photon flux is then calculated as the absorbed photon flux multiplied by the PLQY, η_{PLQY}, which describes the ratio of the number of photons emitted to those absorbed:

$$\Phi_E = \int A(\lambda) d\lambda \cdot \eta_{PLQY} \tag{4}$$

The spectrum of the escaped PL from the bottom of the LSC can then be calculated as:

$$PL_e(\lambda) = \frac{\Phi_E PL(\lambda)}{\int PL(\lambda) d\lambda} \cdot \eta_{out} \tag{5}$$

where $PL(\lambda)$ is the PL spectrum of the luminophore and η_{out} is the the outcoupling efficiency describing the proportion of light that escapes out of the bottom of the film. When the outcoupling efficiency is not explicitly varied, it is equal to $\frac{1}{2}(1 - \eta_{trap})$, where η_{trap} is the trapping efficiency that describes the proportion of light trapped inside the waveguide. η_{trap} is equal to 0.75 because the refractive indices for the glass and the polymer in an LSC are both assumed to be 1.5. The emitted PL spectrum is then converted back to spectral irradiance units (W/m^2) prior to its use in the final transmission calculation:

$$T(\lambda) = \exp(-\alpha(\lambda)\tau c) + PL_e(\lambda) \tag{6}$$

$T(\lambda)$ was then multiplied by 0.92 to account for the reflection of light off of the QD film deposited on a glass pane. Due to the significant influence of light absorption in QD films with high concentrations, we omit the influence of high QD loading on the film's refractive index that would reduce this reflection factor.

Color Fraction Calculation

To calculate the color fractions for blue (F_{BI}), green (F_{GI}), red (F_{RI}), and far-red light (F_{FRI}), the following equations were used:

$$F_{BI} = \frac{\int_{400}^{500} T(\lambda) d\lambda}{\int_{400}^{750} T(\lambda) d\lambda} \tag{7}$$

$$F_{GI} = \frac{\int_{500}^{600} T(\lambda) d\lambda}{\int_{400}^{750} T(\lambda) d\lambda} \tag{8}$$

$$F_{RI} = \frac{\int_{600}^{700} T(\lambda) d\lambda}{\int_{400}^{750} T(\lambda) d\lambda} \tag{9}$$

$$F_{FRI} = \frac{\int_{700}^{750} T(\lambda) d\lambda}{\int_{400}^{750} T(\lambda) d\lambda} \tag{10}$$

The color fractions were assumed to remain constant throughout the lettuce growing cycle.

Day Light Calculation

To simulate both daytime and nighttime while controlling for the extended daily light integral (eDLI), sunrise was assumed to occur at 6 am and sunset was assumed to occur at 6 pm. The intensity of light was calculated in a sinusoidal fashion using the following equation:[16]

$$I(t) = \sin\left(\frac{\pi(t-6)}{18-6}\right) \tag{11}$$

where t is time from 6 am to 6 pm. Outside of this range of time, $I(t)$ is equal to 0 to simulate nighttime. Moonlight was not considered in this work.

For a constant eDLI, $I(t)$ was modulated to meet the target eDLI. Because the lettuce growth model requires light intensity in units of W/m^2, $I(t)$ was converted using the following

equation:

$$I_{ePAR} = \frac{I(t)}{\int_0^{24} I(t)dt} \cdot \frac{eDLI_{target} \cdot 1 \times 10^6}{3600 \cdot 4.6} \tag{12}$$

where $eDLI_{target}$ is the target constant eDLI in units of $mol \cdot m^{-2} \cdot day^{-1}$, 1×10^6 converts the unit of mol to μmol, 3600 converts the units of hour to second, and 4.6 converts the unit of μmol$/m^2/s$ to W/m^2 for light in the ePAR range.

To calculate the required outdoor eDLI, the target eDLI was divided by the integrated transmittance of the QD film:

$$eDLI_{outdoor} = \frac{eDLI_{target}}{f_{ePAR}} \tag{13}$$

where f_{ePAR} is the fraction of transmitted light in the ePAR range compared to the incident solar spectrum:

$$f_{ePAR} = \frac{\int_{400}^{750} T(\lambda)d\lambda}{\int_{400}^{750} F(\lambda)d\lambda} \tag{14}$$

Lettuce Growth Model

The calculation of the lettuce plant yield was based on the model developed by Van Henten,[17] which computes hourly plant dry weight. The light use efficiency in Van Henten's model does not consider spectrum, despite lettuce having wavelength-dependent absorptance of light and relative quantum yield (Figure S2), as reported by McCree.[18] The quantum yield for lettuce refers to the the amount of CO_2 fixed per absorbed photon.

As a result, the model provided by Abedi et al. was used to factor in the transmitted spectrum through the QD film.[19] While the development of these models was not the focus of this work, the assumptions and constants used to calculate spectrum-dependent biomass accumulation are briefly described here.

The model developed by Abedi et al. utilized data from 20 experimental trials of lettuce growth under various color ratios controlled primarily through light emitting diodes (LEDs).

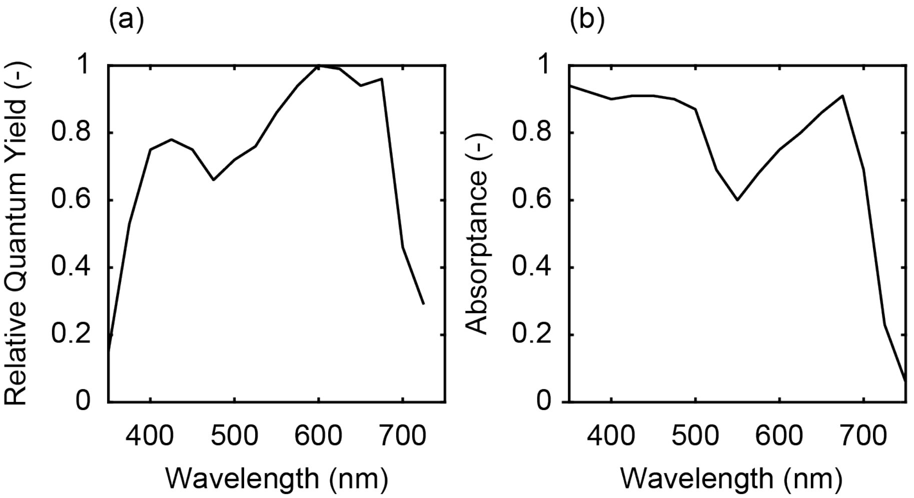

Figure S2: (a) Relative quantum yield and (b) absorptance for lettuce grown in growth chambers retrieved from McCree.[18]

The relationship between the incident color fractions and the resulting lettuce dry weight was then established by varying c_ϵ, the quantum use efficiency, or the energy required to reduce one mole of carbon dioxide. The original model by Van Henten estimated this parameter as 17×10^{-6} g/J and ignored the effect of photon spectral distribution on this parameter. Instead, Abedi et al. provide a regression model to calculate the quantum use efficiency:

$$\begin{aligned} c_\epsilon = R_I[&-1.4 \cdot 10^{-4} + 1.82 \cdot 10^{-4} F_{BI} + 2.06 \cdot 10^{-4} F_{BI}^2 - 7.71 \cdot 10^{-4} F_{BI}^3 + 8.38 \cdot 10^{-4} F_{BI}^4 \\ &+ 2.32 \cdot 10^{-4} F_{GI} - 4.20 \cdot 10^{-4} F_{GI}^2 + 1.53 \cdot 10^{-3} F_{GI}^3 - 1.56 \cdot 10^{-4} F_{GI}^4 \\ &- 1.90 \cdot 10^{-4} F_{RI} + 1.03 \cdot 10^{-3} F_{RI}^2 - 1.10 \cdot 10^{-3} F_{RI}^3 + 4.13 \cdot 10^{-4} F_{RI}^4 \\ &+ 4.68 \cdot 10^{-4} F_{FRI} - 5.25 \cdot 10^{-3} F_{FRI}^2 + 2.92 \cdot 10^{-2} F_{FRI}^3 - 4.77 \cdot 10^{-2} F_{FRI}^4] \end{aligned} \quad (15)$$

where R_I (W/m^2) represents the ratio of spectral irradiance in the ePAR range calculated in the previous section to a reference value, $I_{PAR+FR,R180}$, of 32.8 W/m^2:

$$R_I = \frac{I_{ePAR}}{I_{PAR+FR,R180}} \quad (16)$$

F_{BI}, F_{GI}, F_{RI}, and F_{FRI} are the intensity fractions of blue, green, red, and far-red light,

respectively calculated using Equations 7 - 10:

$$F_{B/G/R/FRI} = \frac{I_{B/G/R/FR}}{I_{ePAR}} \tag{17}$$

All other calculations for estimating biomass accumulation remained the same as those used in Van Henten's model. Van Henten's model estimates the accumulation of dry weight over time as a function of the growing environment. The plant dry weight (g/m^2) considers both structural dry weight (X_{SDW}) and non-structural dry weight (X_{NSDW}) to account for cell walls and cytoplasm as well as glucose, sucrose, and starch, respectively.[20] These two state variables were calculated on an hourly basis:

$$\frac{dX_{NSDW}}{dt} = c_\alpha f_{phot} - r_{gr} X_{SDW} - f_{resp} - \frac{1 - c_\beta}{c_\beta} r_{gr} X_{SDW} \tag{18}$$

$$\frac{dX_{SDW}}{dt} = r_{gr} X_{SDW} \tag{19}$$

where c_α (unitless) converts assimilated CO_2 into carbon, f_{phot} (g/(m^2 · s)) represents the total plant canopy gross photosynthetic rate, r_{gr} (s^{-1}) represents the specific growth rate obeying the Michaelis Menten equation,[20] f_{resp} (g/(m^2 · s)) represents the maintenance respiration of the crop, and c_β (unitless) is a factor for respiratory and synthesis losses due to growth. Constant parameters used in these calculations are listed in Table S3.

Photosynthesis at the canopy was calculated as:

$$f_{phot} = [1 - \exp(-c_K c_{lar}(1 - c_\tau) X_{SDW})] f_{photmax} \tag{20}$$

where c_K (unitless) represents the extinction coefficient for a planophile canopy, c_{lar} (m^2/g) represents the structural leaf area ratio, and c_τ (unitless) represents the ratio of root dry weight to total dry weight. $f_{photmax}$ (g/(m^2 · s)) represents the gross carbon dioxide assimi-

lation rate of the canopy and was calculated as:

$$f_{photmax} = \frac{\epsilon U_{ePAR} g_{CO_2} c_\omega (U_{CO_2} - \Gamma)}{\epsilon U_{ePAR} + g_{CO_2} c_\omega (U_{CO2} - \Gamma)} \tag{21}$$

where ϵ (g/J) represents the light use efficiency, U_{ePAR} (W/m^2) represents the ePAR light intensity calculated in the previous section, g_{CO_2} (m/s) represents the canopy conductance to CO_2 diffusion, c_ω (g/m^3) represents the density of CO_2, U_{CO_2} (ppm) represents the CO_2 concentration in the greenhouse, and Γ (ppm) represents the CO_2 compensation point. The light use efficiency was calculated as:

$$\epsilon = c_\epsilon \frac{U_{CO_2} - \Gamma}{U_{CO_2} + 2\Gamma} \tag{22}$$

Because the CO_2 compensation point is affected by temperature, it can be calculated as:

$$\Gamma = c_\Gamma c_{Q10,\Gamma}^{(U_T - 20)/10} \tag{23}$$

where c_Γ (ppm) represents the CO_2 compensation point at 20 °C, $c_{Q10,\Gamma}$ (unitless) represents the Q10 value which accounts for the effect of temperature, and U_T represents the vegetable temperature (°C). During the day, the vegetable temperature was assumed as 24 °C and at night, the vegetable temperature was assumed as 19 °C.

To calculate the canopy conductance for diffusion of CO_2 from the air to the chloroplasts, the following equation was used:

$$\frac{1}{g_{CO_2}} = \frac{1}{g_{bnd}} + \frac{1}{g_{stm}} + \frac{1}{g_{car}} \tag{24}$$

where g_{bnd} (m/s) represents the boundary layer conductance, g_{stm} (m/s) represents the stomatal conductance, and g_{car} (m/s) represents the carboxylation conductance. The temperature-

dependent carboxylation conductance was calculated as:

$$g_{car} = -1.32 \cdot 10^{-5} U_T^2 + 5.94 \cdot 10^{-4} U_T - 2.64 \cdot 10^{-3} \tag{25}$$

To calculate the transformation rate of non-structural to structural dry weight, the temperature-dependent specific growth rate was calculated as:

$$r_{gr} = \frac{c_{gr,max} X_{NSDW}}{c_\gamma X_{SDW} + X_{NSDW}} c_{Q10,gr}^{(U_T - 20)/10} \tag{26}$$

where $c_{gr,max}$ (s^{-1}) represents the saturation growth rate at 20 °C, c_γ (unitless) controlled for the role of r_{gr} in the conversion of dry weight, and $c_{Q10,gr}$ (unitless) represents the Q10 factor for growth that relies on temperature.

To consider the energy required for the crop to maintain its cell structure, the maintenance respiration was computed as:

$$f_{resp} = [c_{resp,sht}(1 - c_\tau) X_{SDW} + c_{resp,rt} c_\tau X_{SDW}] c_{Q10,resp}^{(U_T - 25)/10} \tag{27}$$

where $c_{resp,sht}$ and $c_{resp,rt}$ (s^{-1}) represent the maintenance respiration coefficients for the shoot and root at 25 °C, respectively, and $c_{Q10,resp}$ (unitless) represents the Q10 factor for respiration.

Finally, the non-structural and structural dry weights were calculated as a function of time (t):

$$X_{SDW,t+1} = X_{SDW,t} + \frac{dX_{SDW}}{dt} \tag{28}$$

$$X_{NSDW,t+1} = X_{NSDW,t} + \frac{dX_{NSDW}}{dt} \tag{29}$$

The total dry weight per plant (Y_{DW}) considered both the structural and non-structural

dry weight as well as the planting density, which we assumed was 24 plants per square meter:

$$Y_{DW} = \frac{X_{SDW} + X_{NSDW}}{24} \tag{30}$$

We assumed 35-day harvesting cycles, after which the structural and non-structural dry weights of the plant reveredt to their initial values, as listed in Table S3.

Table S3: Constant inputs for calculating dynamic dry weight.

Variable	Value	Unit
c_α	0.68	-
c_β	0.8	-
c_K	0.9	-
c_{lar}	75×10^{-3}	m^2/g
c_τ	0.15	-
c_ω	1.83×10^{-3}	g/m^3
U_{CO_2}	450	ppm
c_Γ	40	ppm
$c_{Q10,\Gamma}$	2	-
g_{bnd}	0.007	m/s
g_{stm}	0.005	m/s
$c_{gr,max}$	5×10^6	s^{-1}
c_γ	1	-
$c_{Q10,gr}$	1.6	-
$c_{resp,sht}$	3.47×10^{-7}	s^{-1}
$c_{resp,rt}$	1.16×10^{-7}	s^{-1}
c_τ	0.15	-
$c_{Q10,resp}$	2	-
$X_{DW,t=0}$	0.72	g/m^2
$X_{SDW,t=0}$	$0.75(0.72) = 0.54$	g/m^2
$X_{NSDW,t=0}$	$0.25(0.72) = 0.18$	g/m^2

To validate the model employed here, we compared our calculations to experimental growth trials conducted by Kang et al.[21] Kang et al. grew lettuce and sweet basil under CIS/ZnS films of both low and high concentrations, as well as under polyethylene films

without QDs. We assumed their high-concentration film was at a concentration of 2.4 wt%, resulting in resulting in comparable harvested dry weights after a 19-day growth cycle as shown in Figure S3. We also simulated growth under the control polyethylene film at both high and low eDLIs. The high eDLI was reported for the control film in the work by Kang et al. and the low eDLI was that under the CIS/ZnS film. The simulated yield under high eDLI was also comparable to the experimentally reported value. The simulated yield under the control film at low eDLI was slightly higher than that under the shifted spectrum at the same light intensity, aligning with our results reported in Figure 2. Therefore, we assumed that the changes in dry weight as a function of transmitted spectra reported in this work were reliable.

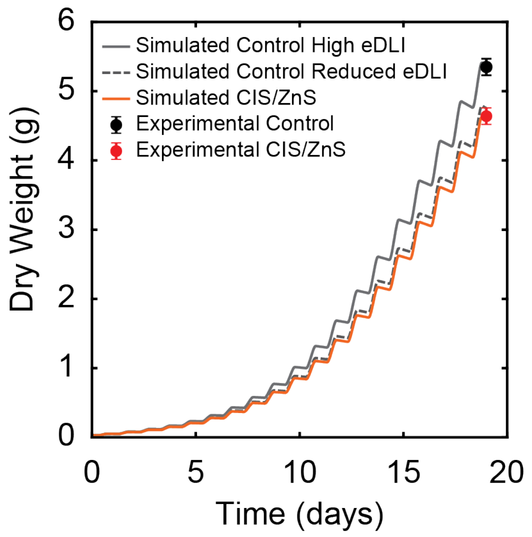

Figure S3: Simulated dry weight of lettuce over a 19-day growth cycle for lettuce grown under control films at both high (gray solid line) and low (dashed solid line) eDLI as well as CIS/ZnS films (orange solid line). Experimental dry weights for lettuce grown under control and CIS/ZnS films from Kang et al. are shown as black and red markers, respectively, at day 19.[21]

Because the biomass accumulation model employed in this work is based on experimental lettuce growth trials, small changes in the incident spectrum could translate to large changes in the expected biomass. For example, lettuce grown under low concentration r-CDot films had a simulated decrease in lettuce dry weight of around 20%, as shown in Figure 2. Despite

the small change in transmitted spectrum, there was a significant change in the simulated biomass accumulation rate. At comparable light intensities and with the same amount of blue light, lettuce growth trials with just 20 μmol m^{-2} s^{-1} less green light and 20 μmol m^{-2} s^{-1} more red light from LEDs (out of a total of around 200 μmol m^{-2} s^{-1}) had a reported change in dry weight from 2.4 g to 1.3 g. The r-CDots were the only QDs that absorbed primarily green light, and thus were the only QDs to exhibit this reduction in lettuce dry weight.

Outdoor eDLI Calculation

To determine the outdoor eDLI across the continental United States, DLI values (400 - 700 nm) were retrieved from Faust and Logan using ArcGIS.[22] These values were spatially resolved to 0.1 °N and 0.1 °W and were reported for each month. To convert these values from DLI to eDLI, they were multiplied by a conversion factor of 1.13 that considered the integrated fraction of light in the solar spectrum from both 400 - 700 nm and 400 - 750 nm. Then, the converted eDLI values were multiplied by a transmittance factor to consider light blocked by greenhouse framing structures if these films were implemented as such. In this work, the greenhouse transmittance was assumed to be 80%.[16] The resulting eDLI maps were created in MATLAB using the contour plot function, `contourfm`.

Additional Supporting Figures

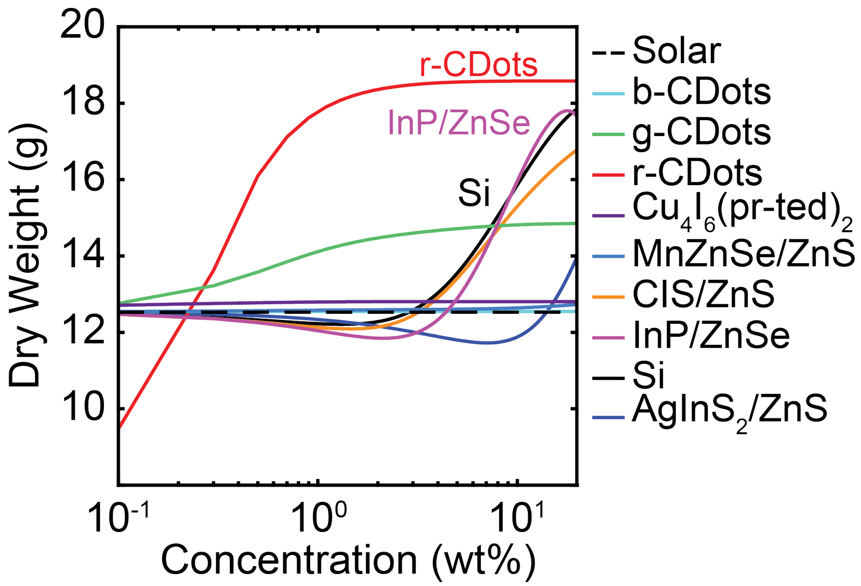

Figure S4: The dry weight of lettuce grown under all nine nontoxic QD films as a function of concentration (colored lines) compared to the dry weight of lettuce grown under the solar spectrum (dashed black line).

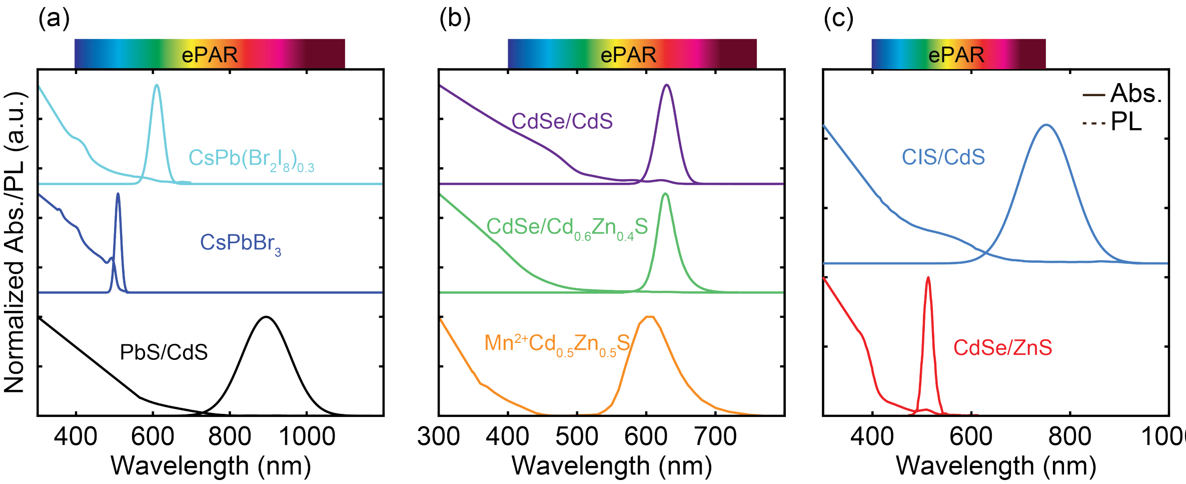

Figure S5: Absorption (solid lines) and photoluminescence spectra (dashed lines) for all QDs studied, organized into (a) Pb-containing QDs, (b) Cd-containing QDs with peak PL wavelengths in the red range, and (c) Cd-containing QDs with peak PL wavelengths outside of the red range. Extended photosynthetically active radiation (ePAR) ranges are highlighted in rainbow.

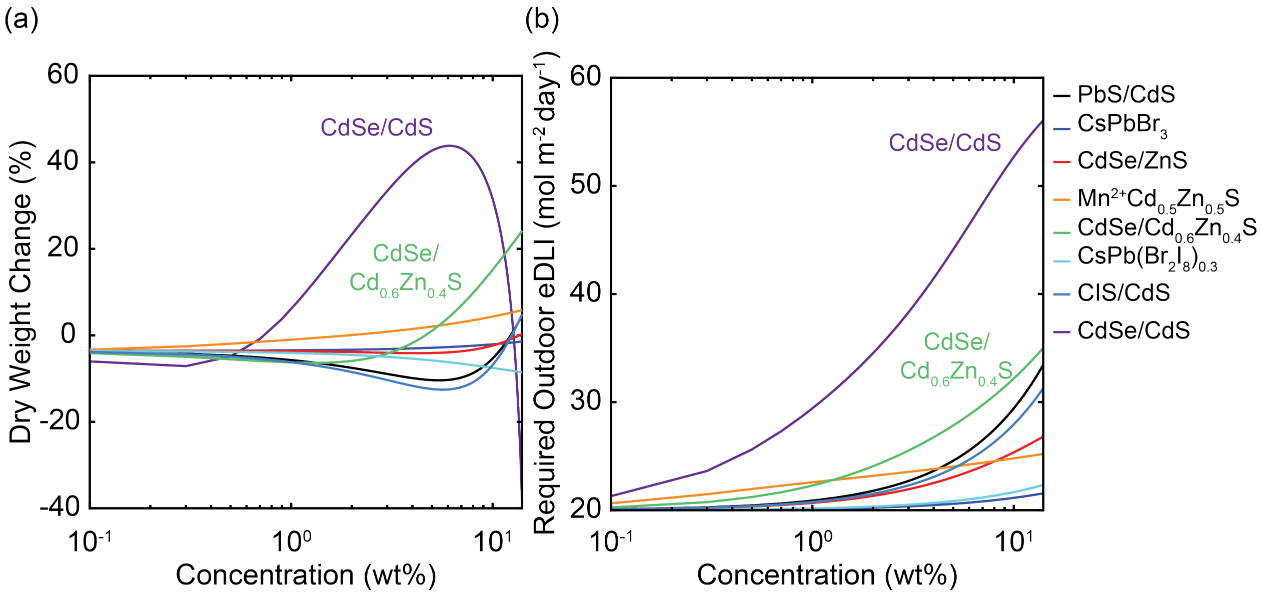

Figure S6: (a) The change in dry weight of lettuce compared to lettuce grown under the solar spectrum (12.5 g) for lettuce grown under Cd- and Pb-containing QD films. (b) Required outdoor eDLI for Cd- and Pb-containing QD films. QD films that resulted in the greatest yield enhancements (CdSe/ZnS and CdSe/$Cd_{0.6}Zn_{0.4}S$) have additional labels.

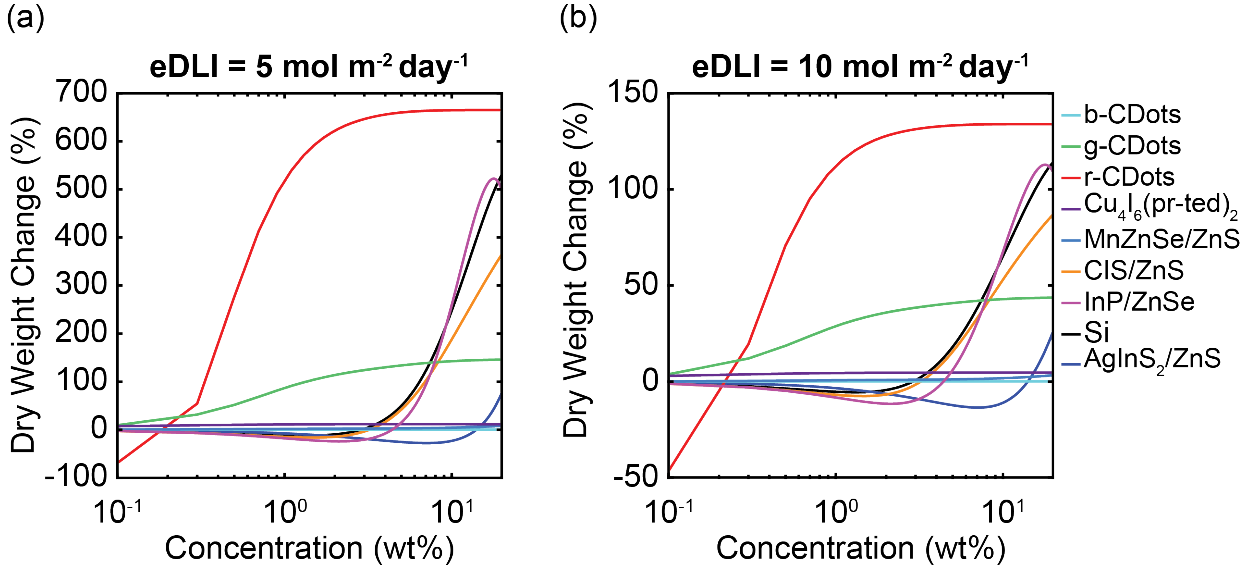

Figure S7: The change in lettuce dry weight as a function of QD concentration in the film at constant eDLI values of (a) 5 $mol \cdot m^{-2} \cdot day^{-1}$ (0.63 g) and (b) 10 $mol \cdot m^{-2} \cdot day^{-1}$ (5.2 g).

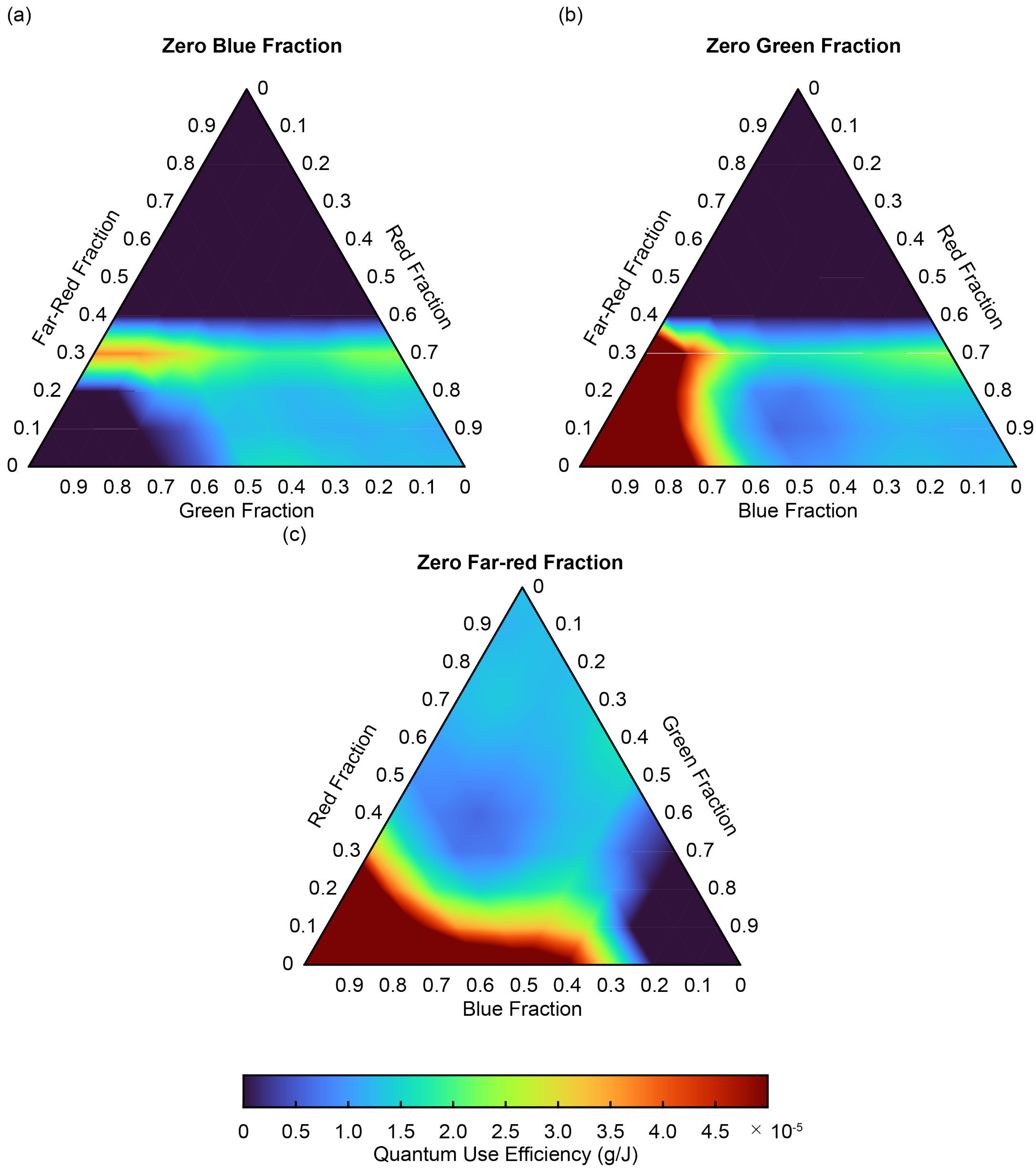

Figure S8: The quantum use efficiency (c_ϵ) for lettuce as a function of blue, green, red, and far-red color fractions assuming (a) F_{BI} is set to 0, (b) F_{GI} is set to 0, and (c) F_{FRI} is set to 0. The source data in the model by Abedi et al. always included some red light, so the red light fraction cannot be set to 0.

References

(1) WebPlotDigitizer. https://github.com/automeris-io/WebPlotDigitizer.

(2) Zdražil, L.; Kalytchuk, S.; Holá, K.; Petr, M.; Zmeškal, O.; Kment, Š.; Rogach, A. L.; Zbořil, R. A Carbon Dot-Based Tandem Luminescent Solar Concentrator. *Nanoscale* **2020**, *12*, 6664–6672.

(3) Parrish, C. H.; Hebert, D.; Jackson, A.; Ramasamy, K.; McDaniel, H.; Giacomelli, G. A.; Bergren, M. R. Optimizing Spectral Quality with Quantum Dots to Enhance Crop Yield in Controlled Environments. *Communications Biology* **2021**, *4*, 1–9.

(4) Erickson, C. S.; Bradshaw, L. R.; McDowall, S.; Gilbertson, J. D.; Gamelin, D. R.; Patrick, D. L. Zero-Reabsorption Doped-Nanocrystal Luminescent Solar Concentrators. *ACS Nano* **2014**, *8*, 3461–3467.

(5) Chen, J.; Zhao, H.; Li, Z.; Zhao, X.; Gong, X. Highly Efficient Tandem Luminescent Solar Concentrators Based on Eco-Friendly Copper Iodide Based Hybrid Nanoparticles and Carbon Dots. *Energy & Environmental Science* **2022**, *15*, 799–805.

(6) Hill, S. K. E.; Connell, R.; Held, J.; Peterson, C.; Francis, L.; Hillmyer, M. A.; Ferry, V. E.; Kortshagen, U. Poly(Methyl Methacrylate) Films with High Concentrations of Silicon Quantum Dots for Visibly Transparent Luminescent Solar Concentrators. *ACS Applied Materials & Interfaces* **2020**, *12*, 4572–4578.

(7) Chen, W.; Li, J.; Liu, P.; Liu, H.; Xia, J.; Li, S.; Wang, D.; Wu, D.; Lu, W.; Sun, X. W.; Wang, K. Heavy Metal Free Nanocrystals with Near Infrared Emission Applying in Luminescent Solar Concentrator. *Solar RRL* **2017**, *1*, 1700041.

(8) Sadeghi, S.; Jalali, H. B.; Srivastava, S. B.; Melikov, R.; Baylam, I.; Sennaroglu, A.;

Nizamoglu, S. High-Performance, Large-Area, and Ecofriendly Luminescent Solar Concentrators Using Copper-Doped InP Quantum Dots. *iScience* **2020**, *23*.

(9) Zhao, H.; Zhou, Y.; Benetti, D.; Ma, D.; Rosei, F. Perovskite Quantum Dots Integrated in Large-Area Luminescent Solar Concentrators. *Nano Energy* **2017**, *37*, 214–223.

(10) Zhou, Y.; Benetti, D.; Fan, Z.; Zhao, H.; Ma, D.; Govorov, A. O.; Vomiero, A.; Rosei, F. Near Infrared, Highly Efficient Luminescent Solar Concentrators. *Advanced Energy Materials* **2016**, *6*, 1501913.

(11) Connell, R.; Keil, J.; Peterson, C.; Hillmyer, M. A.; Ferry, V. E. CdSe/CdS–Poly(Cyclohexylethylene) Thin Film Luminescent Solar Concentrators. *APL Materials* **2019**, *7*, 101123.

(12) Li, H.; Wu, K.; Lim, J.; Song, H.-J.; Klimov, V. I. Doctor-Blade Deposition of Quantum Dots onto Standard Window Glass for Low-Loss Large-Area Luminescent Solar Concentrators. *Nature Energy* **2016**, *1*, 1–9.

(13) Wu, K.; Li, H.; Klimov, V. I. Tandem Luminescent Solar Concentrators Based on Engineered Quantum Dots. *Nature Photonics* **2018**, *12*, 105–110.

(14) Sumner, R.; Eiselt, S.; Kilburn, T. B.; Erickson, C.; Carlson, B.; Gamelin, D. R.; McDowall, S.; Patrick, D. L. Analysis of Optical Losses in High-Efficiency CuInS2-Based Nanocrystal Luminescent Solar Concentrators: Balancing Absorption versus Scattering. *The Journal of Physical Chemistry C* **2017**, *121*, 3252–3260.

(15) Brennan, L. J.; Purcell-Milton, F.; McKenna, B.; Watson, T. M.; Gun'ko, Y. K.; Evans, R. C. Large Area Quantum Dot Luminescent Solar Concentrators for Use with Dye-Sensitised Solar Cells. *Journal of Materials Chemistry A* **2018**, *6*, 2671–2680.

(16) L. D. Albright; A.-J. Both; A. J. Chiu Controlling Greenhouse Light to a Consistent Daily Integral. *Transactions of the ASAE* **2000**, *43*, 421–431.

(17) Van Henten, E. J. Validation of a Dynamic Lettuce Growth Model for Greenhouse Climate Control. *Agricultural Systems* **1994**, *45*, 55–72.

(18) McCree, K. J. The Action Spectrum, Absorptance and Quantum Yield of Photosynthesis in Crop Plants. *Agricultural Meteorology* **1971**, *9*, 191–216.

(19) Abedi, M.; Tan, X.; Stallknecht, E. J.; Runkle, E. S.; Klausner, J. F.; Murillo, M. S.; Bénard, A. Incorporating the Effect of the Photon Spectrum on Biomass Accumulation of Lettuce Using a Dynamic Growth Model. *Frontiers in Plant Science* **2023**, *14*.

(20) Goudriaan, J.; Van Laar, H. H. *Modelling Potential Crop Growth Processes*; Current Issues in Production Ecology; Springer Netherlands: Dordrecht, 1994; Vol. 2.

(21) Kang, S.; Parrish, C. H.; Hebert, D.; Zhen, S. Luminescent Quantum Dot Films Increase the Radiation Capture and Yield of Lettuce and Sweet Basil Compared to a Traditional/Neutral-density Greenhouse Glazing. *HortScience* **2024**, *59*, 988–996.

(22) Faust, J. E.; Logan, J. Daily Light Integral: A Research Review and High-resolution Maps of the United States. *HortScience* **2018**, *53*, 1250–1257.
